# Supplementary material for: Switchable unidirectional emissions from hydrogel gratings with integrated carbon quantum dots
Source: Nat Commun. 2024 Jan 29;15:845. doi: 10.1038/s41467-024-45284-1 (PMC10825124; doi:10.1038/s41467-024-45284-1)
Supplement: Supplementary file 1 — Supplementary Information [file 41467_2024_45284_MOESM1_ESM.pdf]

Supplementary Information

Switchable unidirectional emissions from hydrogel gratings with integrated carbon quantum dots

Chenjie Dai[1], *Shuai Wan*[1], *Zhe Li*[1], *Yangyang Shi*[1], *Shuang Zhang*[2, #],

and Zhongyang Li[1,3,4,5, *]

[1]*Electronic Information School, Wuhan University, Wuhan 430072, China*

[2]*Department of Physics, The University of Hong Kong, Hong Kong 999077, China*

[3]*Wuhan Institute of Quantum Technology, Wuhan 430206, China*

[4]*School of Microelectronics, Wuhan University, Wuhan 430072, China*

[5]*Suzhou institute of Wuhan University, Suzhou 215123, China*

[#]*Email: shuzhang@hku.hk*

**Email: zhongyangli@whu.edu.cn*

Inventory of Supplementary Information

Suppl. Note 1. Hydrogel gratings with different initial film thicknesses and their optical properties

Suppl. Note 2. Optical micrographs of the hydrogel grating under excitation

Suppl. Note 3. Experimentally measured and theoretically calculated order distributions of PL emission

Suppl. Note 4. Theoretically calculated reflection and emission order distributions

Suppl. Note 5. Enhancement and divergence angle of the directional emission

Suppl. Note 6. Simulation for imitating the hydrogel inflation

Suppl. Note 7. Measured angle-resolved PL pattern with different hydrogel gratings

Suppl. Note 8. Characteristics of the PL emission from different period gratings

Suppl. Note 9. Measured far-field angular dispersion of PL emission for s-polarization and p-polarization

Suppl. Note 10. Cycle measurements of humidity-driven tunable PL emission

Suppl. Note 11. Humidity-responsive tunable absorption for Q-IHN

Suppl. Note 12. Measured and simulated absorption of Q-IHN at the normal incidence under different conditions

Suppl. Note 13. Electric field distribution of Q-IHN with different hydrogel thicknesses

Suppl. Note 14. Comparison of emission and reflection peaks

Suppl. Note 1. Hydrogel gratings with different initial film thicknesses and their optical properties

Supplementary Figure 1a shows the thickness profile of the fabricated Q-HIGs from the spin-coated film with different initial thicknesses of 515 nm (sample #1) and 625 nm (sample #2). The initial thickness difference leads to the thickness profile variation due to the different shrinkage heights. To study the impact of structural differences on optical properties, the angle-resolved reflections of hydrogel gratings are measured from sample #1 and sample #2 (Supplementary Figure 1b). It can be observed that the reflection order disappears near the wavelength of 575-610 nm (sample #2), which indicates that the grating ability for steering light is weak at the wavelengths (Fig. 2f). Since the optical response is highly related to the nanostructure parameter, the reflection spectra could be further optimized by designing the structure parameter and improving the fabrication precision.

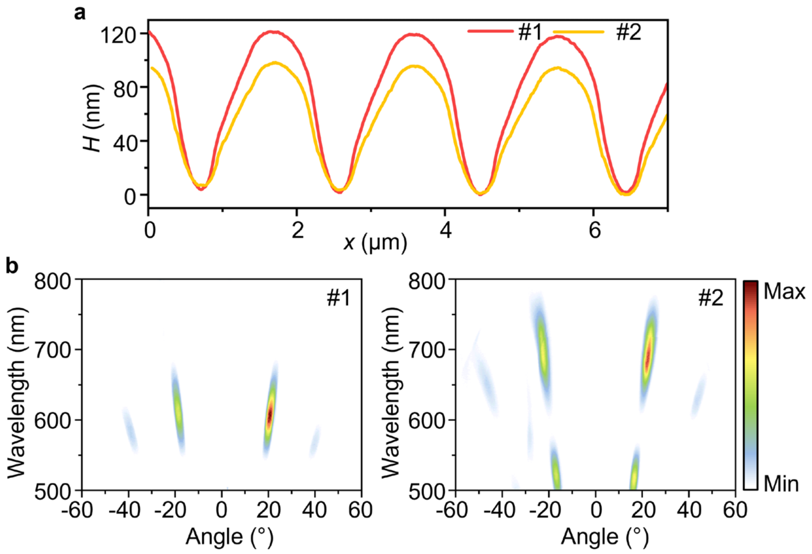

Supplementary Figure 1. Optical properties of different hydrogel gratings. a Measured the thickness profile of the 1800-nm period gratings with the different initial film thicknesses t_1 = 515 nm (red) and t_2 = 625 nm (yellow). **b** Measured broadband angle-resolved reflection of the corresponding hydrogel gratings in (a) with structure parameter difference.

Suppl. Note 2. Optical micrographs of the hydrogel grating under excitation

The captured optical micrographs in Fig. 3a show that there are some visible lines outside the grating under the pump illumination. It can be observed that the visible lines come from PL intensity variation in the film and follow the grating period. This is because the grating is fabricated by the hydrogel shrinkage, and thus the film thickness is higher than the grating. When the grating is obliquely illuminated by the 532 nm laser, part of the light is coupled into the film at the end surface along the contours of the film and grating, as shown in Supplementary Figure 2. Therefore, the additional coupled pump light will propagate over a distance in the film and excite fluorescence light with a grating profile.

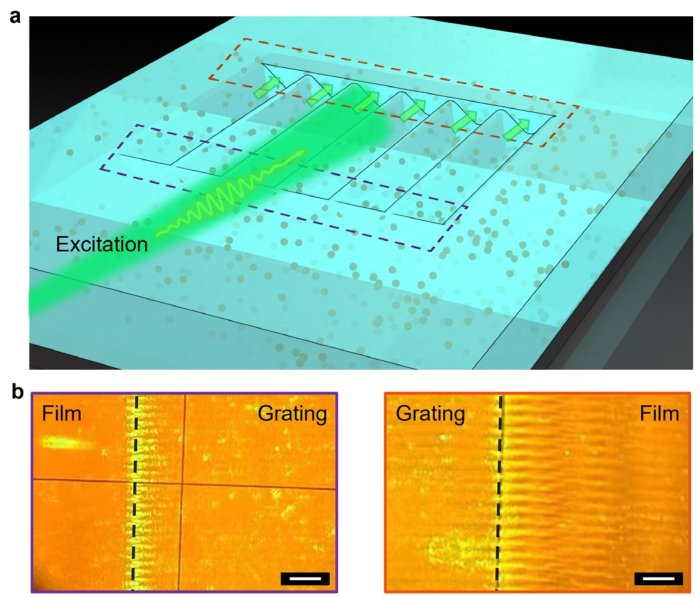

Supplementary Figure 2. Schematic illustration of excitation conditions. **a** Schematic of the fabricated grating on the hydrogel film. **b** Top view of the corresponding area of the grating under the 532-nm pump excitation. Scale bar, 5 μm.

Suppl. Note 3. Experimentally measured and theoretically calculated order distributions of PL emission

To gain more insight into the directional emission, we theoretically calculate the PL order distribution from the grating $\mathbf{k}_G$ and in-plane momentum $\mathbf{k}_{//}$ accumulation, where the emission angle could be expressed as

$$\theta = \arcsin\left(\left(\pm\mathbf{k}_{//} + \mathbf{k}_{\mathrm{G}}\right)/k_0\right) \approx \arcsin\left(\pm n_{\mathrm{PVA}}\sqrt{1-(q\lambda/2n_{\mathrm{PVA}}h)^2} + m\lambda/P\right) \quad (1)$$

Supplementary Figure 3 shows the well-aligned measured/calculated order distribution of PL from the 1800-nm period grating. The thickness of PVA h is set as 625 nm according to the measurement, and the refractive index of PVA n_{PVA} is retrieved to 1.52. It can be observed that the measured PL order is well-aligned with the theoretical calculation and the guided PL is mainly steered by the grating momentum of the second order. Because the guided emission has multiple modes and differences in the grating and thin film, the far-field PL distribution exhibits additional low-intensity diffraction orders whose modes are not dominant.

Since wavelength λ, period P, and refractive index n_{PVA} are constants, the divergence angle of PL $\Delta\theta_{\mathrm{PL}}$ is major determined by the divergence angle of the incident light $\Delta\theta_{\mathrm{in}}$. In another word, the narrow divergence angle of PL emission in this work originates from the high directional guided-mode PL in hydrogel film. In addition, the diffraction pattern could also be affected by the phase gradient deviation from fabrication and aperture diffraction from finite sample area.

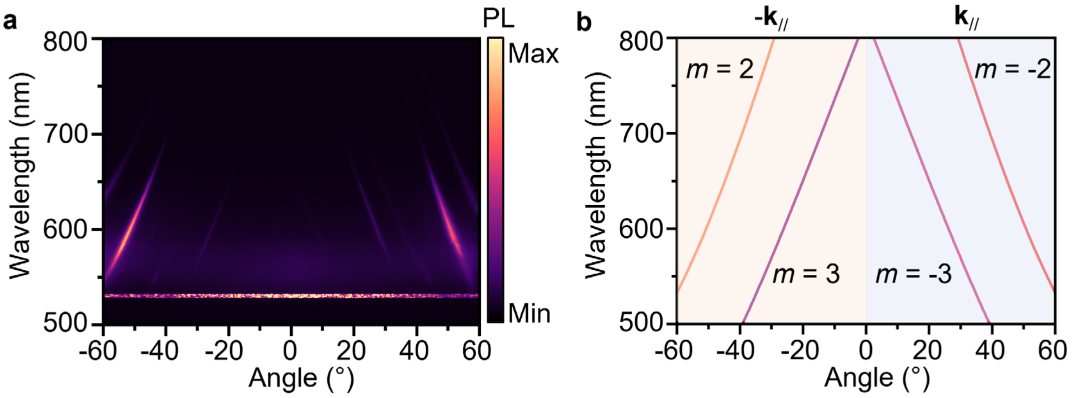

Supplementary Figure 3. Order distributions of PL emission. a Experimentally measured far-field angular dispersion of PL emission from Q-HIG. **b** Theoretically calculated the PL diffraction order distribution based on the momentum equation. The mode number q is set as 1.

Suppl. Note 4. Theoretically calculated reflection and emission order distributions

Supplementary Figure 4 shows the diffraction order relevance between the reflection and PL emission, where the gray area indicates the wavevector component that cannot be collected by 100× objective. Compared to the free-space reflection under normal incident, the guided PL light with an in-plane wavevector $\mathbf{k}_{//}$ induces the diffraction angle shift when coupled into the air. Specifically, the reflection order $m = \pm 1$ (dark blue/red) would be shifted out of the observable range under the PL condition due to the in-plane momentum accumulation. Therefore, although the diffraction efficiency of $m = \pm 1$ is higher than $m = \pm 2$ in reflection, only the diffraction angle of $m = \pm 2$ and ± 3 satisfy the collection condition during PL measurement. In addition, because the diffraction efficiency of $m = \pm 3$ is lower than $m = \pm 2$, the diffraction order $m = \pm 2$ is the dominant order of PL emission in the far field.

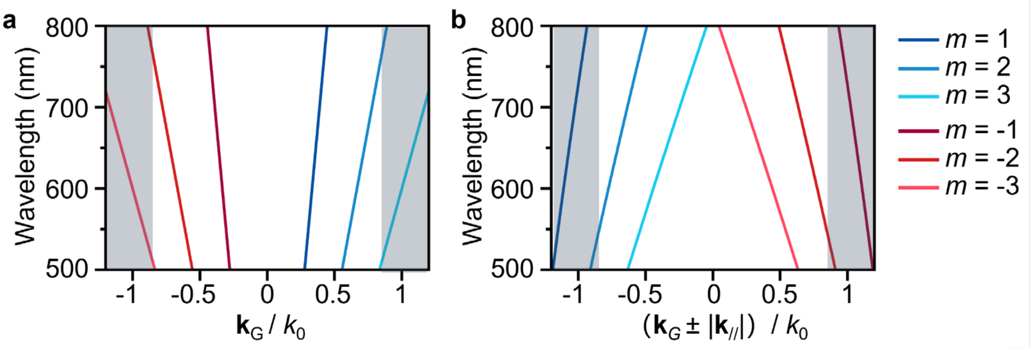

Supplementary Figure 4. Calculated order distribution. Theoretically calculated (**a**) reflection and (**b**) PL order distribution of the hydrogel grating with an 1800-nm period based on the momentum equation.

Suppl. Note 5. Enhancement and divergence angle of the directional emission

Supplementary Figure 5 shows the ±2nd order enhancement and divergence angle of PL from grating at broadband frequencies, which indicates ≈10-fold directional enhancement compared to the thin film. The slight difference between the ±2nd order is due to the asymmetry of the structure and fabrication divergence.

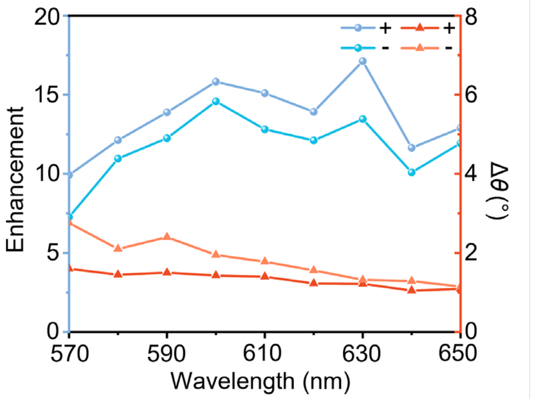

Supplementary Figure 5. Optical characteristics of directional PL emission. Calculated the ±2nd order enhancement of PL from grating and the divergence angle $\Delta\theta$ of the directional PL emission.

Suppl. Note 6. Simulation for imitating the hydrogel inflation

To demonstrate the grating efficiency alteration from hydrogel morphology change, we numerically simulate the beam steering performance of grating under the different RH conditions. Based on our previous measurement, the hydrogel film swells longitudinally while the hydrogel structure mainly expands transversely. The lateral expansion of grating results in a reduction in the phase efficiency, which leads to the disappearance of the far-field diffraction (Supplementary Figure 6). To better visualize the order intensity change, we set the intensity value of the near-zero order to zero.

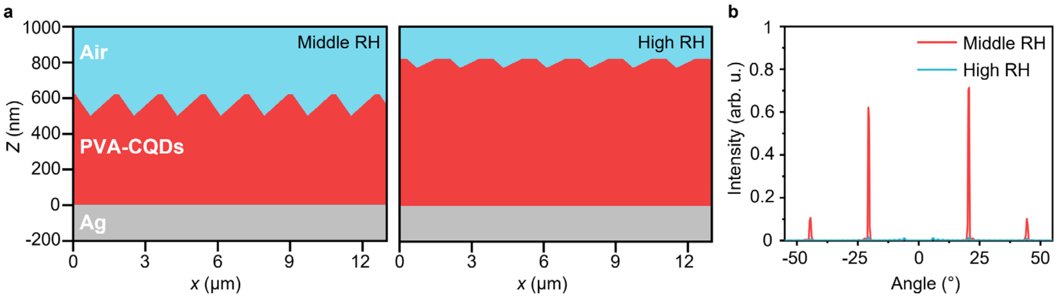

Supplementary Figure 6. Simulation of hydrogel grating under different RH. a Hypothetical schematics for the Q-HIG morphological alteration under different RH conditions. **b** Simulated corresponding angle-resolved reflection intensity change at the normal incidence.

Suppl. Note 7. Measured angle-resolved PL pattern with different hydrogel gratings

Since the intrinsic emission direction in the thin film is also defined by the local density of optical states (LDOS), the momentum of LDOS could be modified by the grating or photonic crystal effect[1,2]. However, the LDOS of emission from an unpatterned film with an initial in-plane momentum means that the PL light is guided and propagates in the film when beyond the critical angle. Therefore, we explain the phenomenon of emission peak by guided mode since the hydrogel grating is fabricated on thin film[3]. The structural parameter difference would induce the guided mode and the local density of optical states variation in the hydrogel film and result in the dominant order shift of far-field PL emission (Supplementary Figure 7), which may suggest another methodology to modulate PL emission.

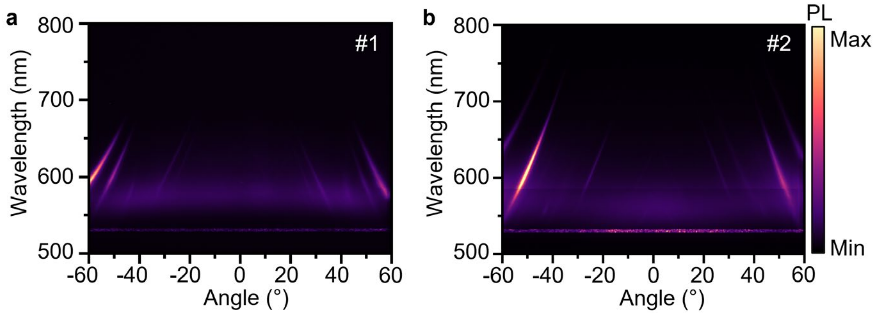

Supplementary Figure 7. Comparison of far-field PL distribution. a, b Measured angle-resolved dispersion diagrams of PL emission from different sample in Supplementary Figure 1.

Suppl. Note 8. Characteristics of the PL emission from different period gratings

Supplementary Figure 8 shows the PL angular distribution of the different period gratings with the initial film thickness of 515 nm at the wavelength of 632 nm. Due to the grating momentum change, the emission angle of PL would shift with the period variation.

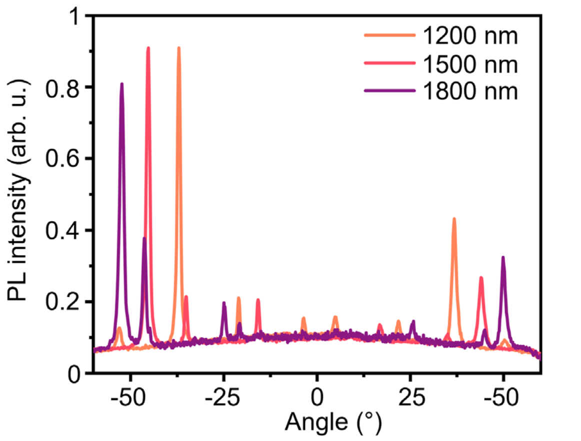

Supplementary Figure 8. Measured angle-resolved PL intensity of the Q-HIGs with different periods at the wavelength of 632 nm.

Suppl. Note 9. Measured far-field angular dispersion of PL emission for s-polarization and p-polarization

Regarding the polarization component of PL emission, Supplementary Figure 9 shows the measured far-field angular dispersion of PL emission for s-polarization (S-pol.) and p-polarization (P-pol.). The P-pol. component is more than ten times weaker than the S-pol. component. However, the enhanced angle-resolved PL distribution of P-pol. indicates that there exists in-plane momentum for P-pol., but it is not dominant due to the 1D grating structure.

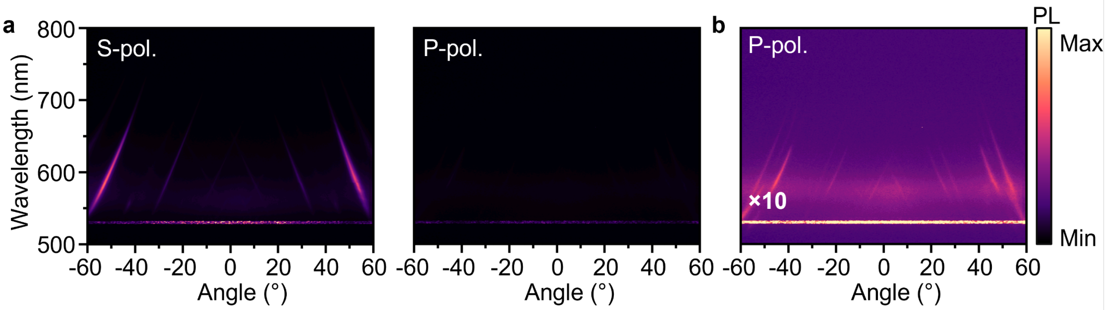

Supplementary Figure 9. Measured PL emission under different polarizatons. a Experimentally measured far-field angular dispersion of PL emission for s-polarization (S-pol.) and p-polarization (P-pol.) using an analyzer. The emission intensities are plotted on the same scale. **b** Measured PL intensity of P-pol. in (a) with ten-fold enhancement.

Suppl. Note 10. Cycle measurements of humidity-driven tunable PL emission

To prove the repeatability of Q-HIG for tunable emission, the cycle measurement of humidifying/drying around the sample between the RH 40-60% (middle RH) and 70-90% (high RH) is conducted to record its PL intensity variation at the diffractive order under the wavelength of 632 nm (Supplementary Figure 10). The PL intensity difference under the same humidity condition is due to the humidity deviation.

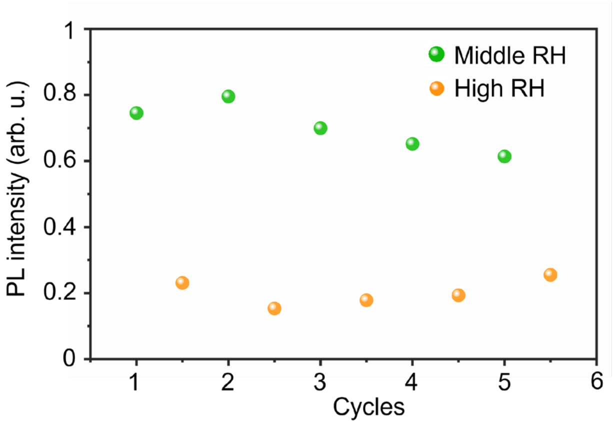

Supplementary Figure 10. The cycle measurements of PL emission control under the RH adjustment.

Suppl. Note 11. Humidity-responsive tunable absorption for Q-IHN

To experimentally prove the tunable angle-dependent absorption from Q-IHN, we fabricate the triple-layered Q-IHN on the silicon substrate. The QDs-integrated hydrogel layer is spin-coated on the bottom Ag mirror, and Ag layers are deposited by thermal evaporation. Due to the angle-dependent absorption, the Q-IHN's photographs exhibit distinct reflected colors under different shooting angles (Supplementary Figure 11a). Supplementary Figure 11b shows the measured angle-resolved reflection of Q-IHN under different humidity conditions and reveals the continuous absorption tuning due to the hydrogel layer inflation.

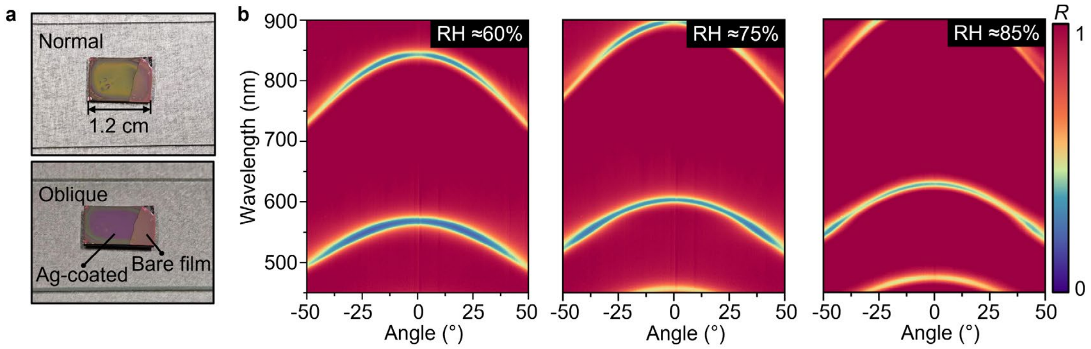

Supplementary Figure 11. Tunable angular absorption. a Top-view photographs of the fabricated Q-IHN and bare QDs-embedded hydrogel film at normal and oblique shooting angles. **b** Measured angle-resolved reflection of the Q-IHN at different humidity conditions.

Suppl. Note 12. Measured and simulated absorption of Q-IHN at the normal incidence under different conditions

The simulated absorption of Q-IHN with varying cavity thicknesses is plotted in Supplementary Figure 12a, which is well-aligned with the measured humidity-responsive absorption alteration (Supplementary Figure 12b). With the RH increasing from ≈60% to ≈85%, the absorption peak wavelength shift exceeds 50 nm, and the average absorption is ≈70%, which confirms the excellent tunable ability from hydrogel inflation. In this design, we fabricate the top Ag layer with a thickness of ≈22 nm to ensure the water molecules exchange, and the absorption could be further enhanced by optimizing the thickness and gas permeability of the top Ag layer.

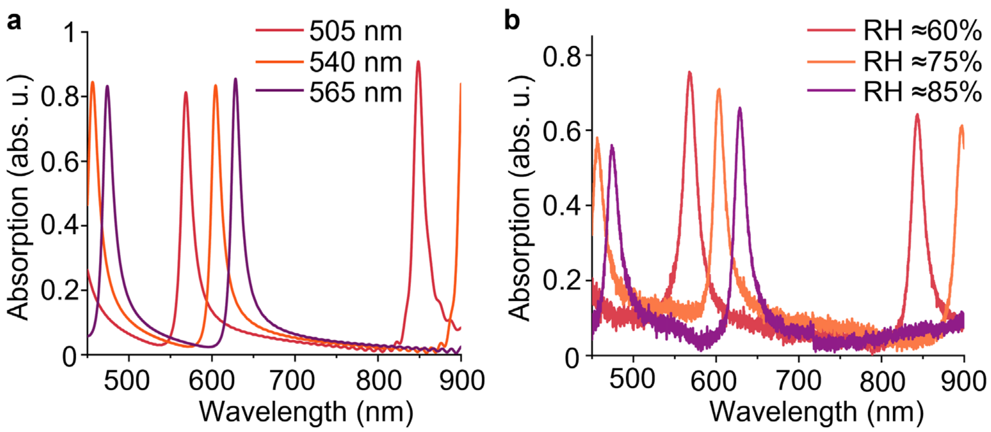

Supplementary Figure 12. Absorption peak shifts from hydrogel inflation. a Simulated absorption of Q-IHN with different hydrogel thicknesses. **b** Corresponding measured absorption of Q-IHN under different RH.

Suppl. Note 13. Electric field distribution of Q-IHN with different hydrogel thicknesses

To visualize the resonance variation in three-layered Q-IHN under different humidity conditions, we calculated the electric field intensity profile with the hydrogel thickness of 505 and 565 nm to mimic the hydrogel swelling from RH ≈60% to RH ≈85% (Supplementary Figure 13). The electric field is prominently confined in the hydrogel layer when the resonance wavelength and cavity thickness satisfy the destructive interference condition, resulting in high-performance absorption.

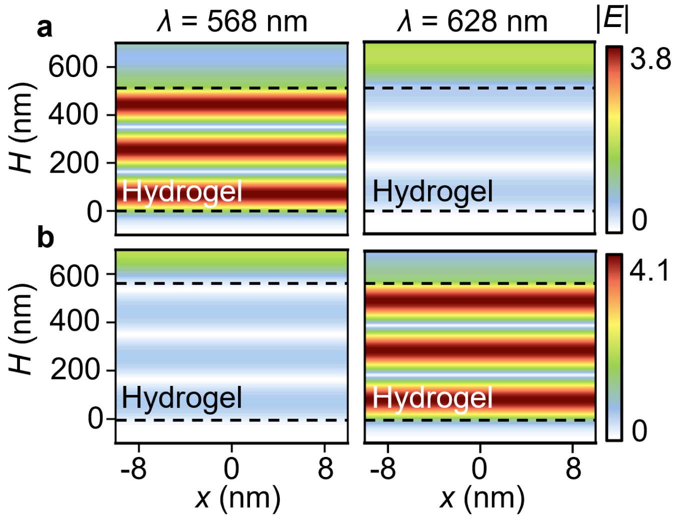

Supplementary Figure 13. Simulated electric field distribution. Electric field distribution of Q-INH with different hydrogel thicknesses of (i) H = 505 and (ii) 565 nm at the absorption peak wavelength of 568 and 628 nm.

Suppl. Note 14. Comparison of emission and reflection peaks

Supplementary Figure 14b shows the corresponding reflection shift of the Q-IHN at the wavelength of 575 nm, and the reflection dips are in excellent agreement with the angular emission peaks (Supplementary Figure 14a) due to the cavity-induced resonance.

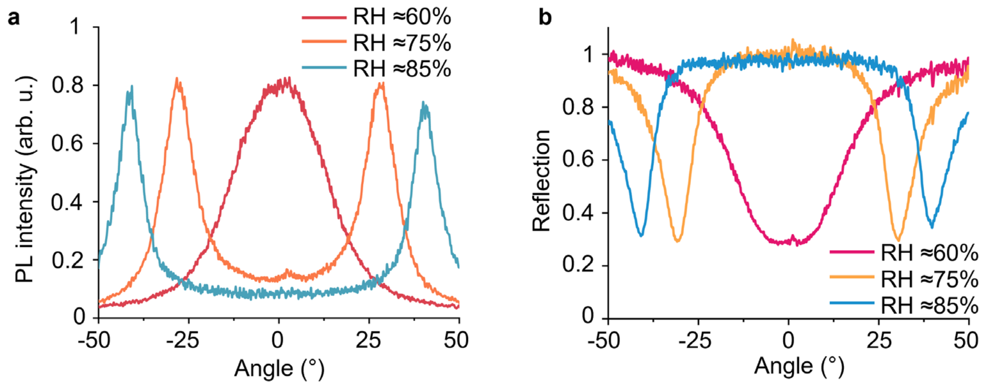

Supplementary Figure 14. Measured angular PL emission and corresponding reflection spectra. a Line plots of the corresponding angle-resolved PL intensity in Fig. 6b at the wavelength of 575 nm, as marked by dashed lines. **b** Corresponding measured angle-resolved reflection of Q-IHN under different RH conditions.

Supplementary References

1 Iyer, P. P. *et al.* Unidirectional luminescence from InGaN/GaN quantum-well metasurfaces. *Nat. Photonics* **14**, 543-548 (2020).

2 Iyer, P. P. *et al.* Sub-picosecond steering of ultrafast incoherent emission from semiconductor metasurfaces. *Nat. Photonics* **17**, 588-593 (2023).

3 Wierer Jr, J. J., David, A. & Megens, M. M. III-nitride photonic-crystal light-emitting diodes with high extraction efficiency. *Nat. Photonics* **3**, 163-169 (2009).
